# Implementation of the Nagoya Protocol in Livestock Sector: What Have We Learnt So Far?

**DOI:** 10.3390/ani11082354

**Published:** 2021-08-09

**Authors:** Elzbieta Martyniuk, Aleksandra Haska

**Affiliations:** Department of Animal Genetics and Conservation, Institute of Animal Sciences, Warsaw University of Life Sciences, Ciszewskiego 8, 02-786 Warsaw, Poland; aleksanda_haska@sggw.edu.pl

**Keywords:** Nagoya Protocol, livestock sector, access and benefit sharing, IRCC, animal breeding and research

## Abstract

**Simple Summary:**

Adoption of the Nagoya Protocol on Access to Genetic Resources and the Fair and Equitable Sharing of Benefits Arising from their Utilization to the Convention on Biological Diversity (Nagoya Protocol) in 2010, and its entering into the force October 2014, created many questions within the genetic resources (GR) sector. The Protocol addresses access to and benefit sharing (ABS) resulting from utilization of GR and associated traditional knowledge (TK). The Protocol has been tailored to address commercial utilization of mainly wild species, and especially plants, due to their gene flow from the biodiverse rich countries of the South to countries in the North. Characteristics of the livestock sector differ in many aspects from the plant sector. Most significantly, the gene flow of livestock genetic resources of breeds and lines with high genetic potential for production is the highest between North–North countries and then North–South countries. Therefore, many working in the livestock breeding industry believed that impacts of the implementation of the Protocol would be limited for their sector. The question arises if this prediction is true, and how can we check impacts based on currently available sources of information related to the implementation of the Nagoya Protocol.

**Abstract:**

The aim of the paper was to analyze impacts of seven years of implementation of the Nagoya Protocol on the livestock sector based on available sources of information and literature. Interim National Reports on implementation of the Nagoya Protocol provided by countries and other information available at the ABS Clearing House managed by the Secretariat of the Convention on Biological Diversity, especially Internationally Recognized Certificates of Compliance (IRCC), were analyzed. Moreover, trends in geneflow of breeding products in selected countries, based on the national reports provided to the UN COMATRADE database, have been compared. Analysis from these sources showed limited impact of the implementation of the Nagoya Protocol for livestock breeding and conservation, as out of 2370 IRCC issued by 31 May 2021, only 573 were granted for animal genetic/biological resources including 90 with livestock as the subject matter. Only one IRCC was granted to a foreign user; all other IRCC were issued for domestic users. The intent was to use livestock genetic/biological resources as bioresources for innovation, which should lead to establishment of the Intellectual Property Rights (IPR) with benefits to be shared through the National Competent Authority or for research purposes.

## 1. Introduction

The third objective of the Convention on Biological Diversity (CBD) is the fair and equitable sharing of the benefits arising out of the utilization of genetic resources [1]. The Convention confirms the sovereign rights of Parties to exploit their own resources pursuant to their own environmental policies. Article 15 clearly describes the process to obtain from provider countries legal access to genetic resources through PIC (Prior Informed Consent), and establishment of a contract, mutually agreed terms (MAT) [2]. In spite of the efforts to facilitate implementation of the third objective of the CBD and adoption of the Bonn Guidelines [3], the end of the Twentieth Century resulted in a number of cases of utilization of genetic resources without meeting the benefit sharing provisions, e.g., neem tree, Enola bean, Basmati rice, wild tomato relatives [4,5,6,7] as well as the traditional knowledge associated with these genetic resources [8]. These developments have strengthened the determination of the Like Minded Mega Biodiverse provider countries to negotiate a new international regime on ABS. This resolve and global collaboration resulted in the negotiation and adoption of the Nagoya Protocol in October 2010 by the Parties to the CBD, at their 10th meeting [9].

The Nagoya Protocol contains three building blocks, access to genetic resources, benefit sharing provisions and compliance, both for Parties to the Protocol and users of genetic resources and associated traditional knowledge. The major tools for legal access, Prior Informed Consent and Mutually Agreed Terms remain the same as adopted in the 15th Article of the CBD. However, provider countries are required to register individual transactions with the ABS Clearing-House (https://absch.cbd.int/ accessed on 19 June 2021) (ABS-CH), a specially designed internet-based system for the Nagoya Protocol that automatically generates a unique identifier for each transaction in a document called the Internationally Recognized Certificates of Compliance. The IRCC provides a sufficient proof for the user that genetic resources and associated traditional knowledge, if relevant, were accessed with PIC and benefit sharing provisions were established in MAT, according to domestic legislative, administrative and policy measures of the provider country.

The Nagoya Protocol contains a number of important provisions, some of them highly relevant for this paper. Article 6 requires Parties to establish domestic access and benefit-sharing legislation or regulatory requirements, unless otherwise determined by that Party. Additionally, Parties shall take measures to ensure that traditional knowledge associated with genetic resources held by indigenous and local communities (ILC) is accessed with the PIC or approval and involvement of these ILCs, and that MAT have been established (9, (Article 7)).

Other important provisions are contained in the Article 8 of the Protocol, which provides three specific cases that require consideration by the Parties while developing their national legislative, administrative and policy measures on ABS. Parties shall consider simplified ABS procedures: (a) to create conditions to promote and encourage research which contributes to the conservation and sustainable use of biological diversity; (b) to address cases of present or imminent emergencies that threaten or damage human, animal or plant health and (c) to consider the importance of genetic resources for food and agriculture (GRFA) and their special role for food security. If such simplified procedures are indeed introduced by Parties, they may affect access to farm animal genetic resources (AnGR).

Article 14 lists all information that Parties shall make available to the ABS Clearing-House, as well as potential additional information. Over time, the ABS-CH has been developed following decisions of the Parties to the Protocol, and today it provides a user-friendly system that plays a key role as the legitimate source of country ABS information, supporting users and other interested stakeholders in their research and business activities based on utilization of GR and associated TK. Full implementation of the Article 14 is crucial for the implementation of the entire Protocol.

The Nagoya Protocol was tailored most of all for plant genetic resources, as they have the highest potential for commercialization and were a subject of biopiracy after the Convention on Biological Diversity came into the force. Therefore, the questions were put forward by Tamminen [10]: What is the true scope of the CBD and Nagoya Protocol in terms of AnGR types; do the signatory parties have identifiable patterns for the implementation of their sovereign powers over AnGR; and if so, would it help to understand what types of entitlement claims, access regulations, etc., are indeed relevant? Consideration of these points may provide a more coherent overview of the exchange of AnGRs’ in the post-CBD and post-Nagoya Protocol world.

With seven years of experience in the implementation of the Nagoya Protocol, it is relevant to question and analyze if and how national ABS measures address farm AnGR. It is also worth determining the interest of potential users to access livestock genetic resources, their objectives for utilization of these resources, and potential obstacles to access encountered, during the ABS authorization procedure set out by provider countries. Another question is to better understand the potential impacts of the Nagoya Protocol on the commercial trade of AnGR, that is, what trends in trade have been observed since its entering into force in 2014.

To find answers to these questions, an analysis of information available in the ABS-CH was carried out based on Interim National Reports on the implementation of the Nagoya Protocol (INR) and Internationally Recognized Certificates of Compliance. The selected data on breeding products obtained from the UN COMATRADE database (https://comtrade.un.org/, accessed on 31 May 2021) were also compared.

## 2. Geneflow in AnGR: What Should Be Expected?

Global exchange of livestock can be dived into three phases, each of them with its own characteristics [11]. The first one from prehistoric times until the eighteenth century resulted in the substantial spread of livestock from their centers of domestication into new areas and regions through human migration, warfare, as well as colonization and trade. The second phase, which lasted till the middle of the twentieth century, occurred mainly in Europe and North America. It resulted from advancements in animal breeding methods, successful selection programs, intensification of livestock production and many other technological developments leading to the substantial trade in improved breeding stock between these two regions. The last phase, from the mid-twentieth century onwards, is directly linked to the livestock revolution [12], a phenomenon of rapid increase in the demand for livestock origin products with the further establishment of intensive production systems in developing countries. The intensified gene flow of high performing animal genetic resources was enhanced by improvements in reproduction biotechnology and more affordable air transportation of germinal products between countries and regions.

A number of studies in the past 15 years or so have indicated that the directions of the AnGR geneflow have been stable, while the volume of trade has increased [11,13,14,15]. This geneflow mainly involves dairy cattle, poultry and pigs.

The most important direction of the livestock gene flow is between developed countries, that either run successful national breeding programs or have strong commercial breeding industries; with the flow contributing to both livestock breeding and production. The second main direction of a flow of livestock genetic resources is from developed to developing countries. These genetic resources with high potential to produce eggs, meat and milk are being used in intensive production systems in the South. They are well adapted to indoor production systems, as they were developed and selected in such husbandry conditions.

Gene flow involving livestock among developing countries was found to be at a low level over the past 15 years, but with potential for substantial increases for exchange. However, this gene flow, especially informal cross-border trade, is not documented and might be underestimated. The flow from developing to developed countries is currently not significant from an animal breeding perspective [16]. Moreover, as documented by [17], it is not given that all imported breeds will become established in new countries. For example, imported Maishan pigs (1980′) and Tuli cattle (1990′), have not played an important role in the US livestock sector as was expected. In contrast, the Boer goat, a native South African breed, imported from Australia in 1993 and then New Zealand, was found attractive by American farmers and was established in the United Stated of America (USA) [17].

These examples also show that, in breeds widely used in different parts of the world, the concept of “provider country” is difficult to apply. In these three pre-CBD cases, only Meishan pigs were imported directly from their country of origin, China, while two other breeds came to USA from Australia/New Zealand. At present, the provider country can only be identified in case of local breeds, as commercial breeds usually have complex ancestries.

## 3. General Progress in Implementation of the Protocol

As of the 19 June 2021, the Nagoya Protocol has been ratified by 131 Parties, 45 from Africa, 17 from Central and South America, (Mexico), 26 from Asia, 33 from Europe and 10 from Oceania; 74% of them being developing countries and 26% developed countries (https://absch.cbd.int/ accessed on 19 June 2021).

However, the level of domestic implementation of the provisions of the Protocol remains unsatisfactory (Table 1). While 89% of countries, Parties to the CBD, have established their ABS National Focal Points, only 72 governments (55%) have established Competent National Authority/ies, that, according to Article 13 of the Protocol, “shall, in accordance with applicable national legislative, administrative or policy measures, be responsible for granting access or, as applicable, issuing written evidence that access requirements have been met and be responsible for advising on applicable procedures and requirements for obtaining prior informed consent and entering into mutually agreed terms”. This means that in the absence of a relevant administrative entity, access to genetic resources from countries is not facilitated and potential users are left to find their way through each country’s process of ABS requirements.

Moreover, only 68 governments have managed to publish their legislative, administrative or policy measures in the ABS-CH, and only 22 (less than 17%) have created systems to enable issuing IRCC that provide legal certainty for users of genetic resources and associated traditional knowledge and constitute the best tool for monitoring utilization of genetic resources within national jurisdiction (Article 17 of the Protocol) [9]. Monitoring domestic users’ compliance is at present, in the early stages of development, at only 34 governments’ designated checkpoints in this respect.

While 98 governments submitted their Interim National Reports on the implementation of the Nagoya Protocol, some indicated plans and approaches in development rather than established measures to implement the Protocol at the national level. Accordingly, it has to be underlined that analysis of the ABS-CH is based on information provided by a limited number of Parties to the Nagoya Protocol.

## 4. Approaches to GRFA through National ABS Legislation

The FAO Commission on Genetic Resources for Food and Agriculture (CGRFA) “is the only permanent intergovernmental body that specifically addresses all biological diversity for food and agriculture. It aims to reach international consensus on policies for the sustainable use and conservation of genetic resources for food and agriculture and the fair and equitable sharing of benefits derived from their use” (http://www.fao.org/cgrfa/overview/how-we-work/en/, accessed on 19 June 2021). The Commission has a long history in addressing ABS issues, including negotiations and adoption in 2001 of the International Treaty on Plant Genetic Resources for Food and Agriculture [18], which was later that year approved by the FAO Council and the FAO Conference. The Commission’s work had an impact on the negotiation of the international regime on ABS, and later on, the Commission has made various contributions to the work undertaken under the Nagoya Protocol.

Ratification of the Nagoya Protocol confirms obligations of Parties to develop national ABS legislative, administrative or policy measures. To support countries in fulfilling these obligations, in the context of genetic resources for food and agriculture, as required in Article 8c [9], the Commission, through its Team of Technical and Legal Experts on ABS, have undertaken work to develop a voluntary guidance-like document, the so called ABS Elements, that was adopted in 2016 [19] and in 2019 was extended by the addition of non-prescriptive explanatory notes describing within the context of the ABS Elements, the distinctive features and specific practices of different subsectors of GRFA, including AnGR [20]. This work was based on the outcome of the International Workshop on ABS for GRFA, organized by the FAO in 2018 in collaboration with the Secretariats of the International Treaty and the CBD [21].

The ABS Elements were developed to draw the attention of national legislators to the specific characteristics of GRFA subsectors and to take these aspects into account in implementation of their national ABS measures, as well as domestic utilization of GRFA. They helped to consider exemptions of AnGR and other genetic resources used for food and agriculture from the national access legislation.

The Interim National Reports on implementation of the Nagoya Protocol showed various approaches in addressing access measures to GRFA. Table 2 summarizes responses to the questions 4, 11, 35 and 36 of the INR (https://absch.cbd.int/search/nationalRecords?schema=absNationalReport, accessed on 19 June 2021).

The majority of the 98 counties that submitted their INRs between 2017 and 2020 declared that they either already have, or are developing national ABS legislation. Access measures, already in place or being developed, were reported by 56 countries. However, it has to be noticed that the situation may have changed from the submissions of the INR, e.g., in 2017. A few countries that declared in Q11 that access to their genetic resources was not subject to PIC had not taken a final decision on the matter while submitting their INR, and later on, introduced access measures, e.g., Belarus or Uruguay.

In total, 66 countries considered the special role of GRFA for food security in developing their national ABS legislation, and 23 from of them provided additional information on this matter (Table 2). These countries can be divided into groups, depending on their response to Q 35 and Q 36. The first group comprises countries that have decided to regulate access to their genetic resources, and considered provisions of Article 8c [9] (e.g., Bulgaria, Ethiopia, France, Malta, Mexico, Namibia, Republic of Korea, Spain, Uganda and Vietnam). Some of them, a second group, provided additional explanatory information (e.g., Croatia, India, Kenya and Peru). Such consideration might result in special provisions on facilitated access to GRFA, including AnGR, or even exemption for GRFA from access measures, as in the case for France or Spain.

The third group of countries decided not to regulate access to their genetic resources and perhaps considered the special features and roles of GRFA in their decision-making processes. Such countries included Chechia, Finland, Germany, the Netherlands, Norway, Slovakia and Switzerland, as well as the EU.

Finally, the last group of countries, including Austria, Belgium, Denmark, Estonia, Hungary, Japan, Poland, Portugal, Sweden and the UK, executing their sovereign rights, decided not to require PIC for genetic resources under the Nagoya Protocol. Therefore, it might be assumed that lack of special consideration of the Article 8c [9] during development of their domestic ABS legislation was not relevant, if such a decision was already taken. Only 10 countries that responded no to Q35 provided a rationale for their decision.

In the course of its work on ABS and GRFA, the FAO Commission on Genetic Resources for Food and Agriculture, in 2017, initiated a survey to collect information from its Members on a number of issues related to national implementation of ABS measures, in particular, on “use and exchange practices, relevant voluntary codes of conduct, guidelines and best practices, and/or standards and community protocols, as well as model contractual clauses on ABS specifically addressing GRFA; on prior informed consent or approval and involvement of indigenous and local communities in this process; and their experiences and views on existing practices in the different subsectors with regard to different uses of GRFA to which ABS measures apply as well as experiences with the use of the ABS Elements” [22].

The survey was carried out separately for National Focal Points/National Coordinators (NFP/NC), with 280 individuals from 136 countries responding. Additionally, 146 stakeholder individuals from 69 countries took part in the survey [23]. The survey indicated a fair level of NFPs’/NCs’ awareness about ABS with much higher involvement in ABS policy-related activities of plant subsector’s representatives. The survey showed that respondents often exchange GRFA from different subsectors; for instance, both plants and microbes. A large proportion of stakeholders indicated they exchange GRFA as part of ongoing collaborations rather than as one-off transactions.

The survey also identified an important difference among GRFA sectors with regard to the development of PIC procedures. It seems that PIC was more developed in the aquatic subsector, followed by the plant and animal subsectors, and was almost absent in the forest subsector. The survey indicated that only a very small proportion of OECD (Organisation for Economic Co-operation and Development) countries have established PIC procedures. In 2018, half of all NFP/NC respondents confirmed that ABS measures have been adopted in their countries, but only one-third of them indicated that ABS measures specifically address GRFA.

It seems that countries that specifically addressed GRFA in their ABS measures, often also considered subsector-level specific details, e.g., for animal, forest or aquatic genetic resources.

About half of the respondents taking part in the NFP/NC survey were aware of the existence of the ABS Elements [19] and 40% indicated their usefulness and active use. The key limitation of the survey was the involvement of a relatively small number of IPLCs representatives, as well as low representation from the micro-organism and invertebrate subsectors [23].

In order to better understand various aspects of ABS legislation regarding GRFA, the Commission decided to prepare a survey on access and benefit-sharing country measures accommodating the distinctive features of GRFA and associated TK [24]. From the perspective of the global exchange of AnGR, access measures established by Parties for their local livestock genetic resources/native breeds are most important, as they may hinder trade, especially at the national and regional levels. The survey [24] presents a number of approaches resulting in specific exemptions of GRFA subject matter from ABS measures. They include, for example, the French ABS law that exempts from its scope three categories of GRFA: genetic resources arising from domesticated or cultivated species, those of related wild plant species, and those that are subject to forestry [25]. In Spain, ABS law exempts from its scope specific PGRs, fisheries resources and AnGR (which are governed under other legislation) [26].

Similarly, the ABS law of Bhutan excludes wild and domesticated PGR and AnGR that are managed under other legislation [27]. In Morocco, exemptions are considered for biological material that is “cultivated or bred for use as a model in research and development” [28]. There are also cases of exclusion granted on a discretionary basis by Australia [24]. In India, the ABS decision-making body, the National Biodiversity Authority, requires certain considerations relating to GRFA to be taken into account when processing a foreign application to access India’s biological resources, such as whether the resource is cultivated/domesticated or wild, developed or conserved ex situ, and if they are of high value/importance to the livelihoods of local communities [29].

## 5. AnGR in the IRCC (Analysis of IRCC at ABS-CH)

**Figure 1:**
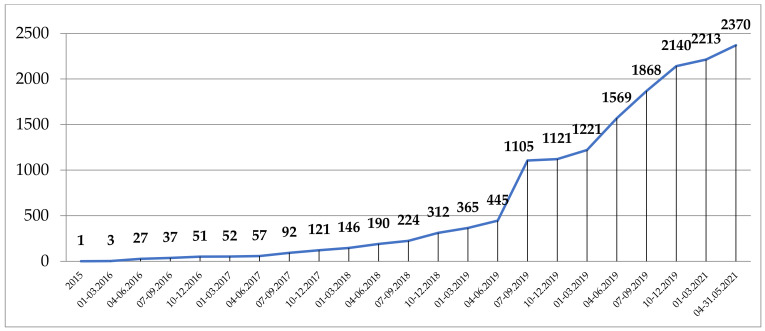
Dynamics of issuing IRCC from 2015 till 31 May 2021.

The first IRCC was issued by the Competent National Authority (CNA) of India, 27 March 2015, for a PhD student at Kent University, UK, for accessing ethno-medicinal knowledge of the Siddi community from Gujarat for research purposes. Since then, the number of IRCC has grown slowly until mid-2019 when the number started increasing up to 660 IRCC issued in the third quarter of 2019, reaching 2370 by 31 May 2021 (Figure 1).

As of 31 May 2021, only 22 Parties provided information to the ABS-CH on IRCC issued for users of their genetic resources and associated traditional knowledge. Parties issuing IRCC, in alphabetical order, include Argentina (1), Belarus (9), Benin (20), Bulgaria (3), the Dominican Republic (2), Ethiopia (1), France (454), Guatemala (2), Guyana (5), India (1521), Kenia (78), the Lao People’s Democratic Republic (11), Malta (8), Mexico (8), Panama (35), Peru (27), the Republic of Korea (10), Saint Kitts and Nevis (2), South Africa (34), Spain (100), Uruguay (3) and Vietnam (36).

According to Avilés-Polanco et al. [30], a relatively high level of biodiversity and the existence of conservation policies in a given country are factors associated with the establishment of both commercial and non-commercial ABS agreements between genetic resource providers and users.

**Figure 2:**
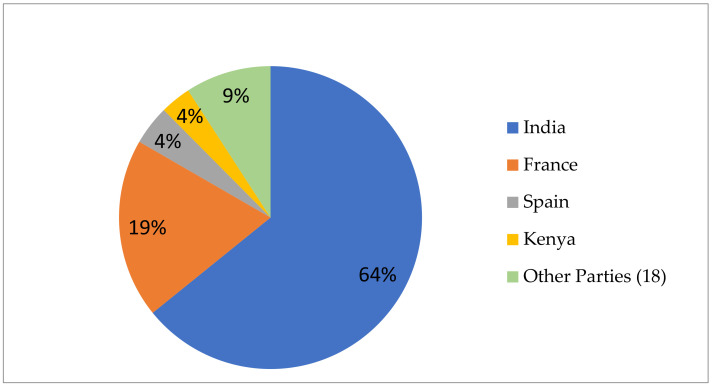
Distribution of IRCC available from the ABS CH as provided by Parties to the Nagoya Protocol, by 31 May 2021.

As is shown in Figure 2 below, the distribution of IRCC issued by the Parties to the Nagoya Protocol regulating access to their genetic resources is extremely uneven. India is highest with 64% of all IRCC issued, and together with France, these two countries were responsible for 83% of all IRCC, while the share of the remaining 20 Parties was only 17%.

It must be stressed that Parties have decided to regulate access not only to international but also to domestic users of genetic resources, which substantially increases the number of IRCC issued. Countries are proceeding with the view that benefits obtained from utilization of domestic genetic resources do not belong only to either international or domestic users, and thus, benefits should be shared with their society or local communities, whatever is most relevant.

An example of this approach is provided by India, where awareness of the importance of protecting innovations by establishing IPR led to the conclusion that biodiversity rich countries should promote local bioprospecting and upgrade local capabilities for IPR [31,32]. India also indicates that potential benefits generated by domestic users through patents should be shared with the society, and relevant IRCCs include such provisions.

**Figure 3:**
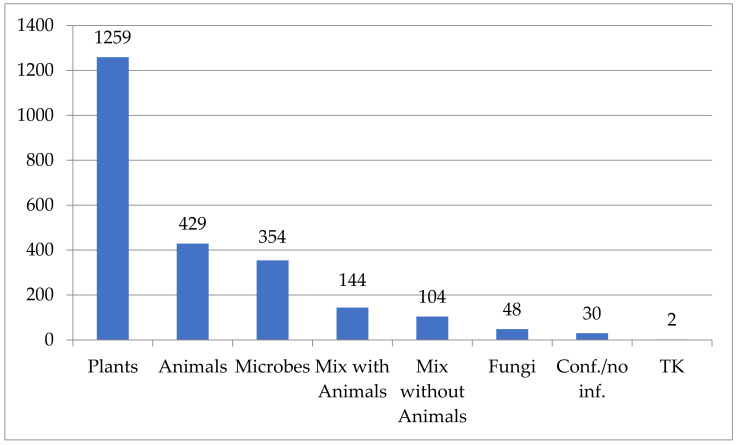
Distribution of genetic resources and traditional knowledge covered by the IRCC, as of 31 May 2021.

Genetic resources that are covered under PIC and MAT and reported in IRCC are mainly plant genetic resources, and were the single subject matter of about 53% of all IRCC issued during the analyzed period (Figure 3). The IRCC issued to access only animal genetic resources constituted 18% of the total IRCC, although 144 of mixed IRCC covering genetic resources belonging to different groups include also animals or their biological material. For instance, the IN-237727-1 subject matter includes plants and invertebrates, whereas IN-250470-1 includes invertebrates, fish, plants and microorganisms. Adding mixed IRCC results in a total 573 IRCC with the AnGR component constitutes 24% of all IRCC. Other mixed IRCC combined other groups of GR. For instance, the BY-255414-1 subject matter includes plants, microorganisms and fungi.

There were two IRCC to access traditional knowledge associated with genetic resources, which may indicate that there are either institutional difficulties in involving IPLC in granting PIC and in negotiating MAT, a low current interest in accessing TK or a lack of awareness of the need to obtain PIC for accessing and using TK.

**Figure 4:**
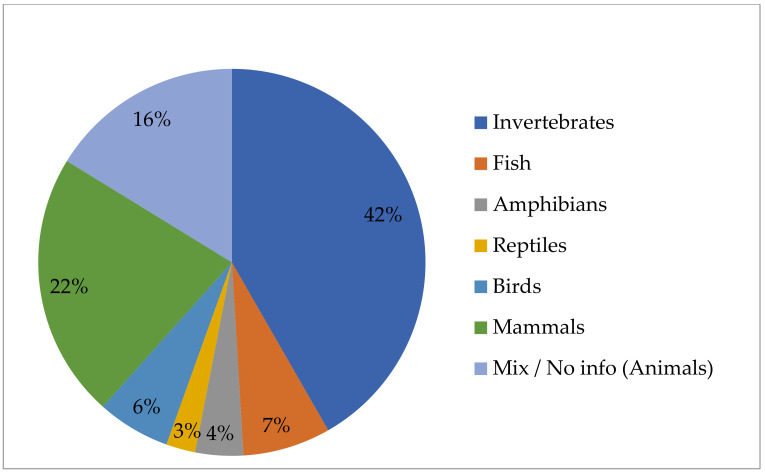
Distribution of IRCC with animal genetic/biological resources, as the subject matter, as of 31 May 2021.

Within 573 IRCC including, as subject matter, animal genetic/biological resources, invertebrates were the most represented group with 239 IRCC, followed by mammals, which accounted for 127 IRCC (Figure 4). The share of birds was much smaller, being the subject matter of 35 IRCC.

As previously indicated, a substantial number of IRCC belong to the mixed group, where access for animal genetic/biological resources was jointly provided with other GR or there was no information on taxonomic classification of animal genetic resources. It included 93 IRCC with an animal mix only and 51 IRCC where the mix included also non-animal taxonomic groups.

**Figure 5:**
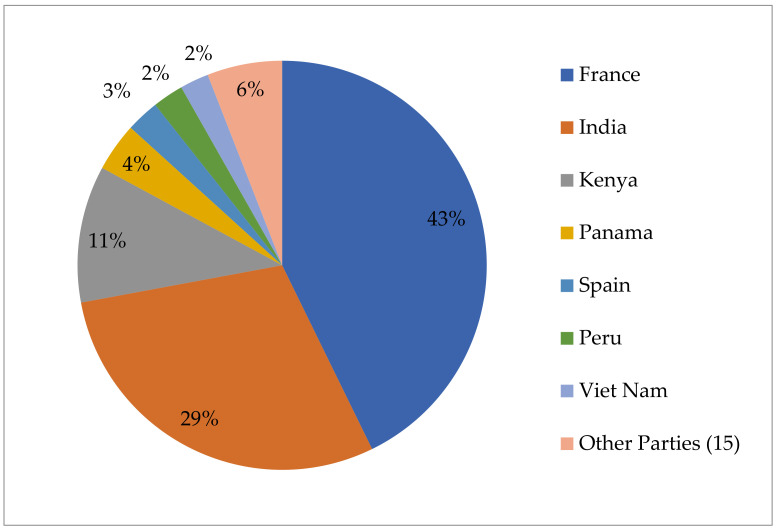
Distribution of animal related IRCC among Parties.

The Party issuing the highest number of 573 animal-related IRCC is France, with 245 IRCC, followed by India (168), Kenya (62), Panama (22), Spain (15), Peru (14) and Vietnam (13). The other 15 Parties jointly have issued 34 IRCC with animal genetic/biological resources being the subject matter (Figure 5).

In total, by 31 May 2021, 90 IRCC (3.8% of the total IRCC) covered farm animal genetic/biological resources, 78 of them from India, 10 from Kenya and one from France and Lao PDR each. The majority of them included not one livestock species, but a few, as well as other genetic or biological resources, e.g., plant or microbial ones.

Only in three IRCC was a specific breed name mentioned, the Jamunapari goat of India (IN-250301-1) as well as Boran, and East African zebu cattle from Kenya (KE-253640-1; KE-253315-1).

Importantly, out of the 90 IRCC that include farm animal species, only one was indeed meeting the expected geneflow, where animal genetic resources from a provider country have been accessed by the user from another country. This one case was from Laos PDR, LA-251864-1, when access was granted to the French Institut de Recherche pour le Développement. The subject matter of this IRCC is described as: “Coronavirus, SARS-CoV2 Origins, insectivorous and frugivorous bats, wild and domestic animals, wildlife markets, Lao PDR”. All other 89 IRCC were granted by the relevant CNA to domestic users.

IRCC granted by Indian and Kenyan CAN differ significantly. In the case of Kenya, all IRCC were for non-commercial activities based on specific research projects, mainly aimed to better characterize farm animal genetic resources, in order, for instance, to use this knowledge in collective breeding to improve genetic gains in dairy cattle breeding programs, or to combat parasites and local diseases.

In India, IRCC were granted to domestic users seeking patents for commercial applications, usually based on a combination of various biological resources. The so called “Bioresources” included animals, their meat and milk, their skins and hairs, specific metabolic derivatives and DNA. The only one application proposing breeding-related patent topic regarded emu sexing. Many proposed innovations addressed various aspects of the leather production process, development of concentrates for animal feeding (where animals were used to test the fodder), preparation of industrial production of Basundi-mix, extraction of animal origin derivatives and their applications in medicine and cosmetic industry (livestock-origin collagen, goat milk to produce soap, silkworm to help wound healing and managing hangovers), just to name a few livestock-related patent development proposals in India.

All domestic users, both institutional and individual, had to be established MAT, and were obliged to share benefits. Usually, if the user “assigns/licenses the process/product/innovation to a third party for commercialization, the user shall pay to the National Biodiversity Authority 3.0% of the fee received (in any form including the license/assignee fee) and 2.0% of the royalty amount received annually from the assignee/licensee”, as required, for instance, in the IN-241038-1 regarding “A novel system effective against cancer and chronic diseases using a temperature controlled bed and a drug” with subject matter of goat meat, sheep meat and cow’s milk.

Such an approach is consistent with the government of India policy to encourage domestic IRP [31,32] but could be considered not to be within the spirt of the Nagoya Protocol and the “grand bargain” between developing and developed countries that led to the formulation of the third objective of the Convention of Biological Diversity.

## 6. AnGR in the International Trade: Examples from the UN COMTRADE

The potential impacts of the implementation of the Nagoya Protocol on commercial trade were analyzed in this paper on the basis of germinal product, bovine semen, which is widely traded worldwide, as well as by comparison of data regarding other breeding products.

**Figure 6:**
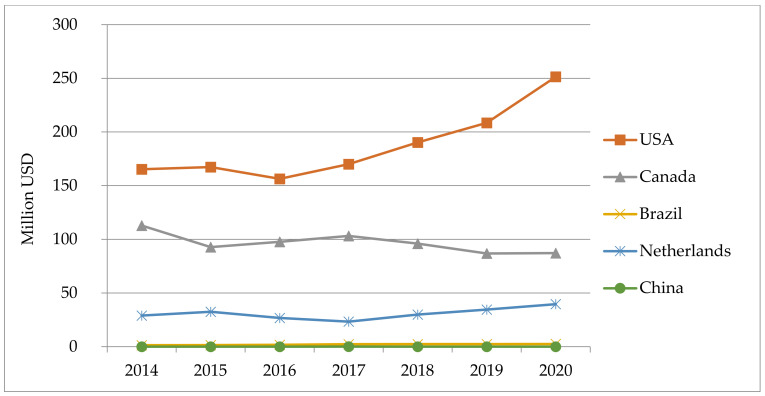
Value of exported bovine semen in selected countries in the period 2014–2020, in USD.

As it is shown in Figure 6, the export value of bovine semen (mainly from bulls with high estimated breeding value) has been substantially increasing for the USA (from 165.305.428 USD in 2014 to 251.421.113 USD in 2020); was oscillating around 90 million USD in Canada, and slightly increasing in this period in the Netherlands, reaching almost 40 million USD in 2020. During this period, the export of bovine semen remained at very low levels in Brazil and China.

**Figure 7:**
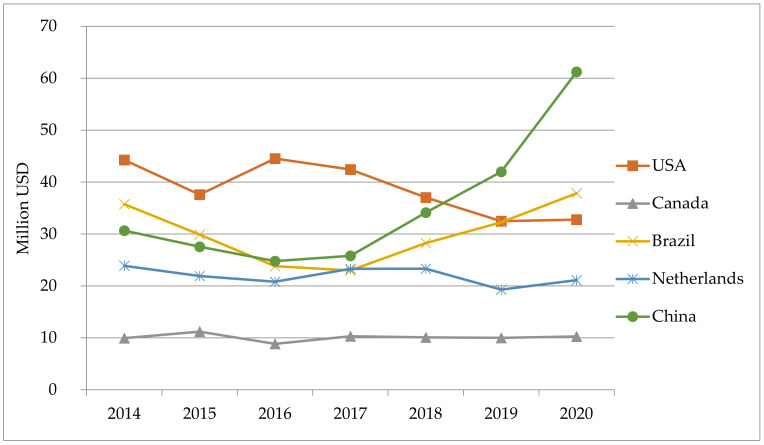
Value of imported bovine semen in selected countries in the period of 2014–2020, in USD.

A different pattern was observed in regard to the import of semen (Figure 7). The semen trade to the USA was decreasing a bit, from over 40 million USD in 2014, to just over 30 million USD in 2020. In Canada, importation was stable at the level of 10 million USD. The major trade in semen is between these two countries.

Semen import to the Netherlands was stable, around 20 million USD, and half in value in comparison to their export. The value of import to China doubled in this period, reaching over 60 million USD in 2020. Brazil, after a sharp decline in 2017, also overturned the level of imports from 2014, reaching almost 38 million USD in 2020.

It can be concluded that the commercial trade of semen has not been affected by the implementation of the Nagoya Protocol, as livestock genetic resources are coming mostly from developed countries, and are owned by breeding industry organizations that sell them on a commercial basis. Many developed countries have not established access measures, or adopted exemptions for GRFA, so even access to their local breeds is without PIC.

It also should be underlined that highly producing genetic resources are sought after by countries that are trying to develop their dairy, poultry or other livestock sectors, e.g., Kenya, Rwanda and India [33,34,35]. The UN COMTRADE database (accessed on 31 May 2020) showed that in 2020, the value of imports to India of live cattle was 2.136.276 USD, of semen, 149.516 USD, and of fowls of Gallus d., below 185g body weight, of 2.964.026 USD. There were no data on exports from India of live cattle or semen and the value of export of Gallus d. chicks was 223.667 USD, with destination to Buthan, Kenya, Uganda and Senegal.

China reported export of fowls of Gallus d., below 185 g body weight, in 2020 at 1.708.267 USD while the importing value of chicks was over 20 times higher, reaching 35.720.694 USD. In the same time, export of chicks from Brazil reached 77.891.366 USD to serve countries in the South American region and Ethiopia. Brazil import value, solely from the UK, was 1.028.102 USD. It shows that commercial trade of genetics for intensive production systems was not hampered, even in countries that have established ABS access legislation, such as Brazil.

The analysis has confirmed earlier observations by Martyniuk et al. [36] that commercial trade of livestock genetic resources has not been adversely affected by the implementation of the Nagoya Protocol and continues to develop, as expected, due to high demand for animal origin products in developing countries [12].

However, commercial trade involving native breeds faces a number of challenges, as, for example, in the case of the request to export germplasm of Ongole cattle from India to Brazil in 2015. The Indian CNA, the National Biodiversity Authority (NBA), decided to constitute an expert committee to take a decision on this matter. The question was, “Should NBA allow for as a South–South arrangement for exchange of germplasm, or may NBA impose restrictions and enter into a benefit sharing agreements and stop third party transfer of developed germplasm from Ongole germplasm?” [37]. In 2018, it was decided to grant permission for the transaction, and Onkole embryos were exported to Brazil [38]. This example shows the complexity that can result in geneflow and the significant amount of time to process applications, even among South-South transfers.

## 7. Impact on Research

It is likely that the impact of implementation of the Nagoya Protocol on livestock research might be substantial in the future, resulting from requirements for administrative approvals in granting PIC. In the case of international breeds, there are established alliances and collaboration among organizations within the breeding industry, which are, currently, outside of ABS implementation processes required under the Nagoya Protocol [39].

As shown by Kenya’s example, PICs have been granted access to biological material of native animals for a number of non-commercial research projects, but the institution that obtained the PIC was either a government agency (Kenya Wildlife Service or Directorate of Veterinary Services, State Department of Livestock Ministry of Agriculture, Livestock and Fisheries) or an academic/research institute (Mpala Research Centre and Mpala Wildlife Foundation). In one case, of IRCC KE-242932-1, valid 2018–2019, with the subject matter of blood and one pin feather obtained from 600 vulturine guinea fowl from Laikipia county, the PIC recipient was declared as confidential. It is therefore hard to predict if applications for research access were submitted by a foreign entity; although the prolonged IRCC for the same subject matter for 2021–2021 has been granted to a domestic research institution.

The experience so far is not encouraging, taking into account different legal circumstances, requirements and ABS practices established in countries that collaborate within international research projects [40]. ABS requirements are adding costs and complexity to already extensive requirements related to veterinary and sanitary OIE regulations and animal identification and registration [41]. Even researches from the provider country are not convinced that ABS measures will positively impact research collaboration, or conservation of domestic genetic resources [42].

As analyzed by Avilés-Polanco et al. [30], of the total number of IRCC issued by 2019, 34% were for commercial purposes, 64% for non-commercial utilization, and 1% declared a potential change of intent. This indicates significantly higher interest in access to genetic resources for research than for other purposes.

## 8. Conclusions

In spite of the fact that 131 governments ratified the Nagoya Protocol, the level of their compliance with the provisions of the Protocol is limited, with 51.9% of Parties having published their ABS legislative, administrative and policy measures, and only 16.8% able to issue IRCC. Additionally, monitoring of domestic users through establishment of the checkpoints is currently only being implemented by 25.9% of governments, mostly by developed countries.

Establishing ABS institutional frameworks, harmonizing existing laws with the new ABS measures, are not an easy tasks, and countries need time to figure out practical and efficient solutions [43,44,45]. A coordinated approach among countries could also prove to be helpful, as demonstrated by the African Union [46]. Educating all actors and stakeholders involved in ABS procedures, too, is very important for effective implementation of the Protocol [27].

In Interim National Reports, 66 out of 98 governments declared that they considered the importance of genetic resources for food and agriculture and their special role for food security; however, there are only a few examples where such consideration resulted in establishment of preferential measures for access to GRFA, including farm AnGR by Parties regulating access to their GR.

While the number of IRCC recorded in the ABS-CH has been growing rapidly over the last two years, reaching 2370 by 31 May 2021, 83% of all of these were issued by only two Parties. Moreover, Parties mostly submitted to the ABS-CH IRCC granted for domestic users, both institutional and individuals. The rapid increase in domestic IRCC could be misleading in terms of assessing the advancement of implementation of the Nagoya Protocol, which was aimed at regulating the international exchange of genetic resources and associated traditional knowledge.

The most common subject matter of the IRCC are plant genetic resources, accounting for 53% of total IRCC. There are also a number of mixed IRCC (10.5%), which include genetic resources belonging to different taxonomic groups.

Animal genetic and biological resources were the subject matter, either as a single one, or in a mix of various animal species, or in a mix with other taxonomic groups in 573 IRCC (24% of all IRCC).

Out of these IRCC, only 90 were related to livestock species, and usually the subject matter covered a number of genetic and biological resources. Only one IRCC included domestic animals granted to a foreign user, and this IRCC was not related to animal breeding.

The analysis of IRCC suggests that there is a limited interest in accessing livestock genetic resources for breeding purposes from countries that are able to issue IRCC, and do not exempt domesticated animals from their ABS measures, like France or Spain [25,26]. However, it is hard to draw conclusions based on examining IRCC only.

As indicated by Pauchard [47], on the basis of the survey from the ABS National Focal Points, there were a lot of agreements taking place between ABS NFP/CNA and users of GR without this being made public. The survey contained three simple questions: How many ABS agreements have been concluded in your country? How many access permits to GR have been issued in your country? If possible, could you indicate the proportion of agreements/permits that have been concluded/issued before and after your country became a State Party to the NP? Through this survey, the information was gathered from states such as Cuba, Costa Rica, Ecuador, Australia, that never registered any transactions using IRCC, but have issued a substantial number of permits. However, it should be noted that the response rate was low, only 24% of countries invited to participate [47].

Therefore, in drawing conclusions based on analysis undertaken in this study, it is important to understand the potential limitations of data available. It would be interesting to analyze AnGR related PIC or other types of permits used by countries that are not yet issuing IRCC and also to better understand the challenges related to implementation of the Nagoya Protocol from AnGR users’ perspective.

Nevertheless, looking at registered trade transactions in UN COMTRADE, we may conclude that the commercial trade in genetic resources of international breeds is blooming, providing benefits for sellers and buyers that can import highly productive genetic material to their countries, and the breeding industry can serve their clients.

On the basis of data analyzed, it seems to that, so far, the Nagoya Protocol has had a limited international impact, if any, on animal breeding and conservation, as livestock related IRCC were granted 99% of the time to domestic users, and the subject matter included mainly biological resources, either for research, as in Kenya, or for development of innovations with the aim to obtain IPR and benefits to be shared with the National Biodiversity Authority, as in India. So far, no IRCC has been granted for farm animal genetic resources to be used in breeding or conservation programs.

## Figures and Tables

**Table 1:** National records available in the ABS Clearing-House (as of 19 June 2021).

Record Types	Number of Records Published	Number of Governments Who Have Published
ABS National Focal Point (NFP)	176	175
Competent National Authority (CNA)	124	72
Legislative, Administrative or Policy Measure	266	68
ABS Procedure	21	17
National Model Contractual Clause	4	4
Internationally Recognized Certificates of Compliance (IRCC)	2383	22
National Websites or Databases	55	41
Checkpoint	71	34
Checkpoint Communiqué	46	6
Interim National Reports on the Implementation of the Nagoya Protocol (INR)	98	98

**Table 2:** Summary of replies to questions related to access measures and GRFA, included in the INR; number of countries and percentage of countries.

Selected Questions from the INR	Responses: Number of Countries (Percentage)					
	Yes	No	Yes/Yes	Yes/No	No/No	No/Yes
4. Has your country taken legislative, administrative and policy measures on ABS?	86 (87.8)	12 (12.2)	n/a			
11. Is access to genetic resources subject to PIC as provided in Article 6.1?	56 (57.1)	42 (42.9)	n/a			
35. In the development and implementation of ABS legislation or regulatory requirements has your country: Considered the importance of genetic resources for food and agriculture and their special role for food security as provided in Article 8 (c)? 36. Additional information	n/a		23 (23.5)	43 (43.9)	22 (22.4)	10 (10.2)

## Data Availability

ABS Clearing-House (https://absch.cbd.int/ accessed on 19 June 2021); UN COMTRADE database (https://comtrade.un.org/, accessed on 19 June 2021).
